# Tangeretin Suppresses Fumonisin Production by Modulating an NmrA- and HSCARG-like Protein in *Fusarium verticillioides*

**DOI:** 10.3390/jof11040313

**Published:** 2025-04-15

**Authors:** Liuqing Wang, Wenlei Zhai, Dongmei Jiang, Nan Jiang, Jiaqi Yan, Haoyun Jiang, Meng Wang

**Affiliations:** 1 Institute of Quality Standard and Testing Technology, Beijing Academy of Agriculture and Forestry Sciences, No. 9 Middle Road of Shuguanghuayuan, Haidian District, Beijing 100097, China; wangliuqing@baafs.net.cn (L.W.); zhaiwl@iqstt.cn (W.Z.); jiangdongmei@baafs.net.cn (D.J.); jiangnan@baafs.net.cn (N.J.); 13240034978@163.com (H.J.); 2 College of Horticulture, China Agricultural University, No. 2 Yuanmingyuan West Road, Haidian District, Beijing 100193, China; yanjiaqi@cau.edu.cn

**Keywords:** fumonisin, mycotoxin, *Fusarium verticillioides*, tangeretin, flavonoid

## Abstract

Fumonisins are polyketide-derived mycotoxins posing significant health threats to humans and animals. Among these, fumonisin B_1_ (FB_1_) is the most prevalent mycotoxin, primarily produced by *Fusarium verticillioides*, especially in maize and its derived products. Tangeretin, a polymethoxyflavonoid, has been identified as having potential medicinal properties, particularly as an antioxidant. To evaluate the antifungal and anti-mycotoxigenic properties of tangeretin and to elucidate the mechanisms underlying its inhibitory effects, assessments of fungal growth, FB_1_ production, conidial germination, and cellulase activity, antioxidant capacity and enzyme activities, transcriptomic analysis and gene deletion experiments were conducted. Consequently, tangeretin significantly curtailed fungal growth and FB_1_ production and provided protection against pathogenic infection on corn. It affected genes associated with fungal growth, conidial development, and antioxidant response. Furthermore, tangeretin interfered with the supply of biosynthetic substrate necessary for fumonisin production, particularly impacting pathways involved in alanine metabolism, pyruvate metabolism, fatty acid degradation, and sphingolipid metabolism. Notably, tangeretin downregulated four biosynthetic genes (*Fum2*, *Fum3*, *Fum10* and *Fum11*) that are involved in the final steps of fumonisin formation. It likely disrupted the MAPK signaling pathway and targeted a putative NmrA- and HSCARG-like protein Fv_Tan1, which was identified as having positive effects on fungal growth and mycotoxin biosynthesis. This study presents a promising approach for controlling fumonisin contamination in agricultural settings.

## 1. Introduction

Fumonisins are linear polyketide-derived mycotoxins primarily produced by various *Fusarium* species [1] and certain *Aspergillus* species [2]. Among these, *F. verticillioides* is a major contributor [3] and a global pathogen responsible for *Fusarium* ear rot in maize [4]. Fumonisin contamination is frequently observed in agricultural products and poses serious health hazards, including neurotoxicity, immunotoxicity, and reproductive toxicity. The International Agency for Research on Cancer (IARC) classifies fumonisins as group 2B human carcinogens [5]. Consequently, maximum allowable levels have been established in many countries and regions [6]. Within this category, fumonisin B_1_ (FB_1_) is the most prevalent and potent, particularly in corn and corn-derived products, significantly escalating health risks [7]. Therefore, developing innovative strategies to effectively control fumonisin contamination is of critical importance.

Although authorized fungicides effectively control plant pathogens, their overuse may lead to chemical residues that threaten human health and promote resistance among phytopathogens. As a result, natural plant extracts have garnered increasing attention as potential substitutes or supplements to synthetic fungicides [8]. These extracts contain bioactive components with medicinal properties, such as anti-inflammatory, antioxidant, and even anti-tumor activities [9]. Many medicinal ingredients, notably phenolic and flavonoid compounds, exhibit antifungal activities [10]. Interestingly, some of these active compounds demonstrate fungicidal activity without anti-mycotoxigenic effects, while others offer both antifungal and anti-mycotoxigenic benefits. By identifying inherent and unique molecular differences through comparative analysis, potential target genes closely associated with mycotoxin regulation can be discovered. This approach is pivotal for screening diverse plant extract ingredients, enabling effective control of mycotoxin contamination.

Phenolic and flavonoid compounds in plant extracts typically induce intracellular changes, affecting oxidative stress balance. Previous studies have shown that various regulators involved in stress responses also play roles in fumonisin biosynthesis. In addition to the specific regulator Fum21 within the fumonisins’ biosynthetic gene cluster [11,12], various external regulators—such as transcription factors, epigenetic modifiers, Mitogen-activated protein kinase (MAPK) cascades, and peroxisomal proteins—play significant roles in responding to external materials and environmental changes while modulating fumonisin biosynthesis [11]. Notably, environmental response transcriptional factors, such as velvet complex [13] and AtfA [14], positively regulate fumonisin biosynthesis. Peroxisomal proteins, including FvPex5 [15] and FvPex8 [16], are involved in maintaining cellular oxidative homeostasis and are implicated in FB_1_ production. Genes within the MAPK signaling pathway are similarly involved in regulating responses to abiotic stress and fumonisin formation. Specifically, FvBck1, a component of the cell wall integrity MAP kinase pathway, is responsible for oxidative stress response in *F. verticillioides* [17], while the HOG-type MAP kinase Fphog1 is involved in nitrogen starvation stress response in *F. proliferatum* [18]. These proteins, through their roles in stress response and fumonisin production, potentially mediate the effects of various active compounds. This mediation may ultimately lead to the downregulation or inhibition of mycotoxin biosynthesis. To our knowledge, no relevant potential targets have been identified for the control of fumonisin in *F. verticillioides* using plant extracts. However, pyrrocidine, derived from maize kernel endophyte *Sarocladium zeae* induces a genetic repressor, FvZBD1, which effectively shuts off fumonisin biosynthesis [19]. In other studies concerning mycotoxin control, perillaldehyde has been found to inhibit *A. flavus* growth and aflatoxin production by disrupting cAMP-PKA signaling regulation [20], although this pathway does not significantly influence FB_1_ biosynthesis [21].

This study identifies tangeretin, a flavonoid renowned for its promising pharmacological effects, including anti-inflammatory, neuroprotective, and anti-cancer properties [22], as a potent dual-functional agent capable of suppressing fungal growth and fumonisin biosynthesis. Tangeretin’s further investigation on controlling fumonisin contamination was also motivated by its medical relevance to neuroprotective potential, which aligns with the urgent need to mitigate fumonisin-induced neurotoxicity in contaminated crops and food products. Further study demonstrates that its inhibition on fumonisin production underscores its role in disrupting redox regulation, restricting biosynthetic substrate supply and directly impacting fumonisin biosynthetic genes. Notably, this research is the first to characterize an NmrA- and HSCARG-like protein as a potential target for tangeretin via the MAPK signaling pathway, exploring its effects on fungal growth and fumonisin production. These inhibitory activities of tangeretin could facilitate the development of alternative agents for controlling fumonisin levels. Furthermore, understanding the mechanisms of tangeretin’s action provides insights into how active substances can counteract pathogenic fungi and suppress mycotoxin biosynthesis.

## 2. Materials and Methods

### 2.1. Screening of Medicinal Compounds for Inhibition Against F. verticillioides

Five medicinal components were tested to determine their effectiveness in suppressing fungal growth against *F. verticillioides*, including tangeretin, resveratrol, curcumin, nobiletin and hesperidin (Yuanye, Shanghai, China). These compounds were separately dissolved into dimethyl sulfoxide (DMSO) and the corresponding concentration was adjusted to 1 mM in potato dextrose agar (PDA) medium. For fungal spores’ production, fresh mycelial plugs were cultured into liquid carboxymethyl cellulose (CMC) medium and collected by centrifuge, which was referred by the method of Tang et al. [23]. The concentration of these spores was standardized to 1 × 10^6^ spores/mL using a hemocytometer. Subsequently, five microliters of this spore suspension were inoculated on the PDA plates and then incubated at 25 °C for 7 days to assess fungal growth and inhibition.

### 2.2. Antifungal and Antimycotoxigenic Properties of Tangeretin

#### 2.2.1. In Vitro Inhibitory Effects of Tangeretin

The inhibitory effects of tangeretin and resveratrol were investigated at a concentration of 1 mM in liquid PDB medium. A spore suspension (1 × 10^6^ spores/mL, *v*/*v* = 1/100) was inoculated into liquid medium with or without chemical treatment and cultured at 25 °C for 7 days with shaking at 180 rpm. For FB_1_ extraction, an equal volume of acetonitrile containing 0.1% formic acid was added to the culture, followed by overnight mixing to ensure complete extraction. After centrifugation at 12,000 rpm for 10 min, 1 mL of the supernatant was filtered through a 0.2 μm polytetrafluoroethylene (PTFE) syringe filter (Pall, Port Washington, NY, USA) for subsequent analysis. FB_1_ quantification was performed using high-performance liquid chromatography with fluorescence detection (HPLC-FLD, Agilent, Santa Clara, CA, USA) after precolumn derivatization. Samples and standards underwent derivatization with o-phthalaldehyde and were injected into HPLC-FLD with an excitation wavelength of 335 nm and an emission wavelength of 440 nm [24]. Chromatographic separation was achieved using a Zorbax RRHD Plus C18 column (3.0 × 50 mm, 1.8 μm; Agilent, Santa Clara, CA, USA) by a gradient elution method, which began with 50% each of solvent A (10 mM KH_2_PO_4_, pH 2.3) and B (methanol:acetonitrile = 1:1, *v*/*v*), shifted to 30% A and 70% B from 3 to 6 min, and returned to the initial conditions from 6.1 to 9 min.

#### 2.2.2. Protective Efficacy of Tangeretin on Corn

To assess the protective efficacy of tangeretin in controlling *F. verticillioides* and its production of fumonisin, fresh corn was utilized. Initially, the fresh corn was sterilized using sodium hypochlorite, followed by thorough drying. To mitigate any potential damage from DMSO, tangeretin was dissolved in edible oil instead. This solution was evenly applied to the surface of the corn to ensure complete absorption for 24 h. Subsequently, fungal spores were inoculated onto the corn surface. The development of pathogenic disease was monitored and documented. Fumonisin production was ultimately extracted and quantified to evaluate the potential practical value of using tangeretin.

#### 2.2.3. Effect of Tangeretin on Conidial Germination of *F. verticillioides*

The impact of tangeretin on the germination rate of *F. verticillioides* conidia was evaluated using a plate assay. A 200 μL aliquot of spore suspension (1 × 10^6^; spores/mL) was uniformly spread on PDA plates containing 1 mM tangeretin, with untreated PDA plates serving as controls. All plates were incubated overnight at 25 °C. Germination rates were calculated from the microscopic observation. Statistical comparisons were made between tangeretin-treated and control groups to assess germination inhibition.

### 2.3. Biochemical Analysis

To investigate the impact of tangeretin on fungal cellular architecture and oxidative stress modulation, mycelia were harvested from cultures grown with and without tangeretin treatment. The structure-related changes were assessed by measuring the enzymatic activity of cellulase as an indicator of structural alterations using Cellulase Activity Assay Kit (Solarbio, Beijing, China). The total antioxidant capacity of the mycelia was evaluated by ABTS (2,2′-azino-bis(3-ethylbenzthiazoline-6-sulfonic acid)) method provided by Total Antioxidant Capacity Assay Kit (Beyotime, Shanghai, China). Reactive oxygen species (ROS) management within the fungal cells was further analysed by measuring hydrogen peroxide (H_2_O_2_) levels and the activities of catalase and total superoxide dismutase (SOD) using respective assay kits (Beyotime, Shanghai, China) following the provided manual instructions. For these assays, the mycelia were grounded and homogenized with lysis buffer in a glass homogenizer using liquid nitrogen. The homogenate was then centrifuged to obtain the supernatant. Protein content in the supernatant was determined by bicinchonininc acid (BCA) method using the Enhanced BCA Protein Assay Kit (Beyotime, Shanghai, China).

### 2.4. Differential Transcriptomic Analyses

To uncover the potential mechanisms underlying tangeretin’s antifungal and antifumonisin effects, mycelia treated with and without tangeretin were subjected to differential transcriptomic analyses. These analyses were carried out by Hangzhou Lianchuan Biotechnology Co., Ltd. (Hangzhou, China). To construct RNA libraries, total RNA was extracted using TRIzol reagent (Thermo Fisher Scientific, Carlsbad, CA, USA) from which high-quality RNA (RIN number > 7.0) was selected for cDNA library construction. The final cDNA libraries were achieved at an average insert size of 300 ± 50 bp. Sequencing was performed on an Illumina Novaseq™ 6000 (Illumina, San Diego, CA, USA) following the recommended protocol. Raw reads were obtained from the sequencing machines. To obtain high quality clean reads, raw reads were further filtered by Cutadapt v1.9 [25]. Subsequently, the quality of these reads was verified using FastQC v0.11.9, which assessed parameters, including Q20, Q30 and the GC-content, of the cleaned data. The sequence data have been deposited in the Genome Sequence Archive [26] in the National Genomics Data Center [27], China National Center for Bioinformation / Beijing Institute of Genomics, and the Chinese Academy of Sciences (GSA: CRA017074) that are publicly accessible at https://ngdc.cncb.ac.cn/gsa accessed on 17 June 2024.

For data analysis, reads from all samples were aligned to the *F. verticillioides* reference genome (https://www.ncbi.nlm.nih.gov/datasets/genome/GCF_000149555.1, accessed on 6 July 2011) using HISAT2 v2.0.4 package [28], which includes initial quality-based read trimming and mapping to the reference genome. Mapped reads were assembled using StringTie v1.3.4d with default parameters [29]. All sample transcriptomes were merged to reconstruct a comprehensive transcriptome using GffCompare v0.9.8 [30]. Expression levels were quantified using StringTie v1.3.4d [29] and Ballgown v3.2 (https://bioconductor.org/packages/release/bioc/html/ballgown.html accessed on 9 March 2025) [31], calculating FPKM (fragment per kilobase of transcript per million mapped reads) values for each transcript. Differential expression analysis was performed by DESeq2 (https://bioconductor.org/packages/release/bioc/html/DESeq2.html accessed on 30 March 2025) [32], identifying genes with a false discovery rate (FDR) below 0.05 and an absolute fold change ≥ 2 as differentially expressed genes (DEGs). These DEGs were then subjected to enrichment analysis of Gene Ontology (GO) functions and Kyoto Encyclopedia of Genes and Genomes (KEGG) pathways to determine their biological impact.

### 2.5. Function Analysis of Potential Target Genes

Pertinent genes identified from the crossover analysis of the differentially expressed genes across treatment groups were considered potential targets of tangeretin. They encode an NmrA- and HSCARG-like protein (FVEG_03055; Fv_Tan1) and a multidrug resistance (MDR) transporter (FVEG_04238; Fv_Tan2). Bioinformatical analyses were separately performed on them, including similarity, conserved domain, and phylogenetic relationship with other homologous proteins from mycotoxin-producing fungi by MEGA11 [33]. The NmrA- and HSCARG-like protein carried out its interaction with intracellular proteins by STRING [34]. The transmembrane helices of the MDR transporter were predicted by TMHMM v2.0 [35].

To investigate their roles in fungal growth and fumonisin production, they were separately knockout by a homologous recombination strategy. The flanking sequences of both genes were amplified separately using the primer pairs mentioned as up-F/R or down-F/R (Table S1). In parallel, hygromycin resistance gene (*HYR*) was also amplified from a plasmid template. Following amplification, the flanking sequences and *HYR* were assembled through nested PCR using the primer pair knock-F/R. The resultant PCR products were purified and used to transform into the protoplast of *F. verticillioides*. Transformants were identified using PCR with two primer sets: up-F/HYR-test-R and in-F/R. The first set was designed to amplify the upstream gene sequence along with a partial *HYR* sequence, confirming gene replacement. The second primer set aimed to amplify part of the internal gene sequence to verify the absence of any residual gene sequence in the chromosomal DNA. Furthermore, transformants were subjected to quantitative PCR (qPCR) analysis to verify the presence of a single, correct homologous recombination event involving the target gene, as referenced in Zhang et al. [36]. Subsequently, the wild-type and mutant strains were cultured in the presence of tangeretin to assess the impact of gene deletion on both growth and mycotoxin production.

### 2.6. Statistical Analysis

All experiments were conducted in triplicate. Data were calculated and expressed as mean ± standard error (S.E.). Significant differences between groups were determined using one-way Analysis of Variance (ANOVA) followed by a post-hoc Tukey test (*p* < 0.05) by IBM SPSS Statistics 26.0 (Armonk, NY, USA). Graphical representations of the data were generated using GraphPad Prism 8.0 (La Jolla, CA, USA).

## 3. Results

### 3.1. Suppression of Fungal Growth and Fumonisin Production by Tangeretin

**Figure 1:**
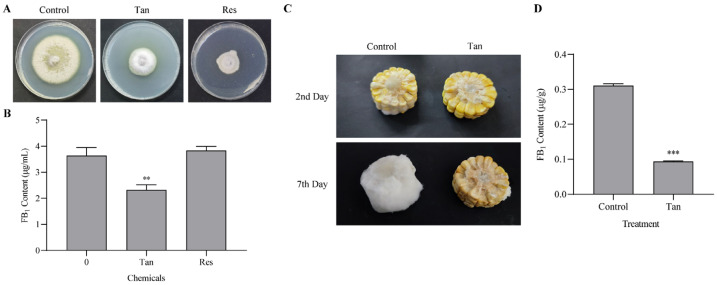
Effects of tangeretin (Tan) and resveratrol (Res) on *Fusarium verticillioides*. (**A**) Fungal growth is suppressed by both tangeretin and resveratrol at a concentration of 1 mM. (**B**) Production of fumonisin B_1_ (FB_1_) is inhibited by tangeretin, while resveratrol does not exhibit a similar effect. (**C**) Protective effects of tangeretin on fresh corn against *F. verticillioides* infection, evidenced by a reduction in infection severity. (**D**) Corn treated with tangeretin shows decreased accumulation of FB_1_. Statistical significance of differences in FB_1_ levels between groups was denoted as ** *p* < 0.01 and *** *p* < 0.001.

In an evaluation of antifungal properties, five medicinal substances were selected for their effects against *F. verticillioides* and *F. graminearum*. Among these compounds, tangeretin demonstrated inhibitory effects on both *Fusarium* species (Figure 1A and Figure S1). Specifically, tangeretin’s inhibition of *F. graminearum* was notably weak, showing a significant inhibition rate of 22.3% (Figure S1). In contrast, *F. verticillioides* showed greater sensitivity to tangeretin with a markedly higher inhibition rate of 46.7%. In addition, resveratrol exhibited the highest inhibition against the hyphal extension of *F. verticillioides* (Figure S1). Based on these observations, both tangeretin and resveratrol could be considered for controlling *F. verticillioides* due to their inhibitory effects. Further analysis in liquid PDB medium revealed that 1 mM tangeretin significantly suppressed 36.4% of FB_1_ production. In contrast, resveratrol did not demonstrate a noticeable inhibition of mycotoxin biosynthesis under similar conditions (Figure 1B).

The efficacy of tangeretin was further evaluated on fresh corn to control the growth of *F. verticillioides* and its production of fumonisin. Observations from the second day showed emerging fungal growth that progressively enveloped the entire corn surface by the seventh day. Contrastingly, tangeretin application effectively inhibited the proliferation of hyphae, significantly protecting the corn from fungal colonization (Figure 1C). In the presence of tangeretin, hyphal growth was notably slower and shorter compared to control samples without tangeretin intervention. Conclusively, mycotoxin analysis revealed a substantial 69.8% reduction in FB_1_ production (Figure 1D), underscoring tangeretin’s potential as a protective agent against mycotoxin output. This demonstrates its practical utility in agricultural applications for the control of fumonisin production.

### 3.2. Interference of Tangeretin in Hyphal Extension, Conidial Development and Redox Homeostasis

**Figure 2:**
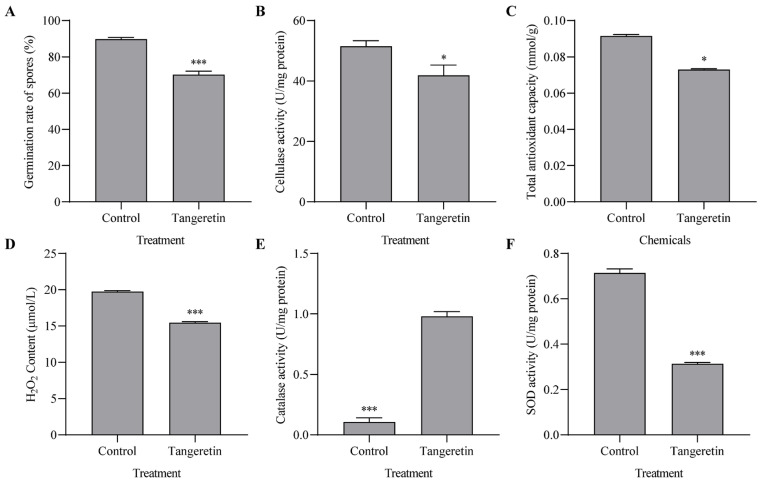
Impact of tangeretin on physiological processes in *F. verticillioides*. (**A**) Conidial germination and (**B**) cellulase activity are inhibited by tangeretin. (**C**) Total antioxidative capacity, (**D**) H_2_O_2_ content, (**E**) catalase activity, and (**F**) superoxide dismutase (SOD) activity related to reactive oxygen species modulation are assessed in *F. verticillioides* treated with tangeretin. Notably, all parameters, except catalase activity, are reduced. Statistical significance of differences in FB_1_ levels between groups was denoted as * *p* < 0.05 and *** *p* < 0.001.

A comprehensive investigation was conducted to explore the influence of tangeretin on the biological activities of *F. verticillioides*, with a specific focus on spore germination and oxidative stress responses. Exposure to 1 mM tangeretin significantly reduced conidial germination rates by 19.5% compared to the control (*p* = 0.0008, Figure 2A). This reduction in germination rates may impede mycelial extension of *F. verticillioides*, thereby potentially limiting fungal proliferation. Further analyses of enzymatic activity revealed significant structural changes, notably a reduction in cellulase activity (Figure 2B). Given that mycelial elongation in fungi largely relies on the hydrolysis and reformation of the cell wall facilitated by cellulase, decreased cellulase activity could directly contribute to the observed suppression of fungal hyphal extension.

Given tangeretin’s potent antioxidant activity documented in previous studies [22,37], the regulation of reactive oxygen species (ROS) was scrutinized. Notably, the total antioxidant capacity of *F. verticillioides* decreased by 20.2% when exposed to tangeretin (Figure 2C). Additionally, the levels of H_2_O_2_ reduced by 21.7% in response to tangeretin exposure (Figure 2D). The activities of the antioxidant enzymes were also evaluated including catalase and SOD. Remarkably, catalase activity significantly increased under tangeretin stress, which was possibly related with the decrease of the observed H_2_O_2_ (Figure 2E), while total SOD activity was notably reduced by 56.08% (Figure 2F). These findings suggest that tangeretin influences the enzymatic activities involved in ROS detoxification within *F. verticillioides*.

### 3.3. Transcriptomic Analysis of F. verticillioides Reveals Tangeretin Impacts

To explore the influence of tangeretin on *F. verticillioides* and its fumonisin production, a differential transcriptomic analysis was performed under tangeretin-treated and untreated conditions (Table S2). In total, 1165 differentially expressed genes (DEGs) were identified, including 297 upregulated and 868 downregulated DEGs, upon exposure to tangeretin (Figure S2).

**Figure 3:**
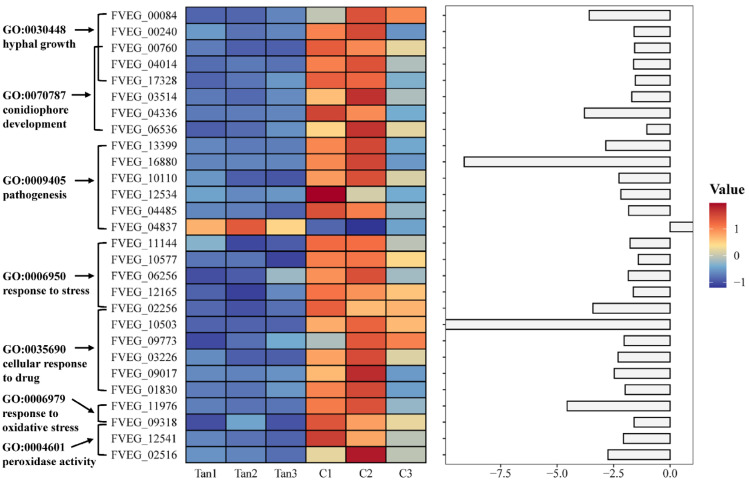
Gene Ontology (GO) functional analysis from the comparative transcriptomes of *F. verticillioides* treated with tangeretin. Differentially expressed genes (DEGs) related to hyphal growth, conidiophore development, pathogenesis, and response to oxidative stress are displayed in a heatmap created using OmicStudio tools at https://www.omicstudio.cn/tool (accessed on 8 June 2023) based on FPKM values.

The statistics of GO and KEGG pathway enrichment were performed to systematically delineate the inhibitory effects of tangeretin on fungal growth and mycotoxin production. Five DEGs enriched into hyphal growth (GO:0030448) were completely downregulated (Figure 3 and Figure S3), correlating with observed reductions in hyphal extension. Of the enriched genes in conidiophore development (GO:0070787), six DEGs were lower transcribed under tangeretin-treated condition, which aligned with the inhibition of conidial germination. In the enrichment of pathogenesis analysis, the transcripts of six DEGs were influenced by tangeretin, including 1 upregulated and 5 downregulated genes. Notably, genes related to stress response (GO:0006950) and cellular response to drugs (GO:0035690) were entirely downregulated when exposed to tangeretin. Moreover, two DEGs enriched in the response to oxidative stress (GO:0006979) were also lower expressed.

**Figure 4:**
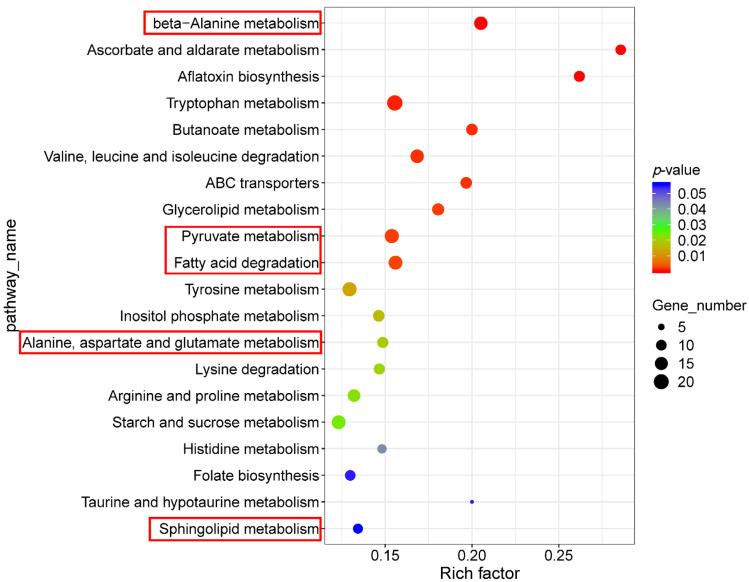
KEGG pathway enrichment analysis from the comparative transcriptome of *F. verticillioides*. Enrichment statistics and corresponding scatter plot are based on *p*-values and gene counts. Pathways associated with fumonisin biosynthesis are highlighted in red boxes.

The KEGG pathway enrichment analysis demonstrated significant enrichment in pathways related to multi-drug resistance, particularly those coding for ABC transporters, highlighting a stress response that potentially initiates resistance mechanisms against abiotic stress. Relevant metabolic pathways, including beta-alanine metabolism (ko00410) and alanine, aspartate and glutamate metabolism (ko00250) enriched, were crucial for fumonisin biosynthesis (Figure 4). Alanine is one of the substrates from the chemical structure, which is condensed with a linear 18-carbon-long polyketide to form the fumonisin backbone [38]. In addition, pathways such as pyruvate metabolism (ko00620) and fatty acid degradation (ko00071) were enriched, which were integral to the synthesis of key fumonisin precursors and components. The metabolism of fumonisin analogue sphingolipid (ko00600) was also enriched in the KEGG pathway, suggesting that tangeretin could possibly affect sphingolipid metabolism, ultimately reducing fumonisin production.

### 3.4. Effects of Tangeretin on Fumonisin Biosynthetic Gene Cluster

**Figure 5:**
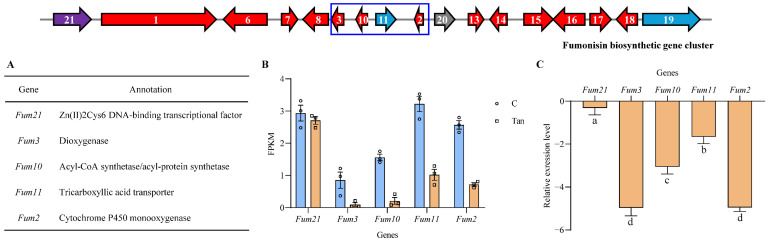
Analysis of DEGs within the fumonisin biosynthetic gene cluster. (**A**) Details of the specific regulator Fum21 and four differential expressed proteins (Fum3, Fum10, Fum11, and Fum2) are shown. The fumonisin biosynthetic gene cluster is depicted in the figure according to Li et al. [11], with numerical labels corresponding to specific *Fum* gene nomenclature. Color-coded arrows denote distinct functional categories: purple arrow represents a regulatory gene; red arrows indicate enzymatic genes; blue arrows symbolize transporter genes; and gray arrow designates one gene with uncharacterized function. (**B**) Transcription levels of *Fum21* along with four DEGs are presented based on RNA-Seq data. Genes *Fum3*, *Fum10*, *Fum11*, and *Fum2* are significantly downregulated by tangeretin. (**C**) The expression levels of five genes in *F. verticillioides* treated with and without tangeretin stress was further validated by RT-qPCR.

Exposure to tangeretin significantly alters the expressions of four genes within this cluster—*Fum2*, *Fum3*, *Fum10* and *Fum11*—resulting in reduced activity compared to the stress-free control (Figure 5A). The relative expression levels of three chosen cluster genes were further confirmed by RT-qPCR (Figure 5B). *Fum2* and *Fum3* encode a cytochrome P450 mono-oxygenase and a dioxygenase catalyzing the hydroxylation at C-10 and C-5 [39,40]. An acyl-CoA synthetase/acyl-protein synthetase Fum10 is responsible for the formation of tricarboxylic CoA to supply two side chains at C-14 and C-15 [11], which requires the transport of tricarboxylic acid by a tricarboxylate transporter protein encoded by *Fum11* [41]. Overall, this demonstrates that tangeretin could directly influence the biosynthetic gene cluster and crucial steps in the biosynthesis of fumonisin, particularly the formation of tricarboxylic ester and two terminal steps of fumonisin formation.

### 3.5. Identification of Tangeretin’s Potential Target Genes in F. verticillioides

**Figure 6:**
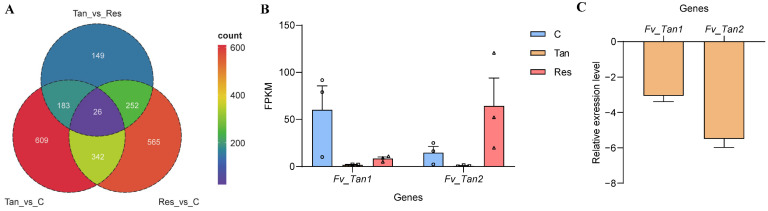
Analysis of potential tangeretin-responsive target genes in *F. verticillioides*. (**A**) A Venn diagram depicting the DEGs from comparative groups treated with tangeretin, resveratrol, or control. (**B**) Transcription analysis of *FVEG_03055* (designated as *Fv_Tan1*) and *FVEG_04238* (designated as *Fv_Tan2*), which encode an NmrA- and HSCARG-like protein and a multidrug resistance transporter, respectively, is presented. Symbols: circle, square, and triangle correspond to the FPKM values of the control group, tangeretin-treated group, and resveratrol-treated group, respectively. (**C**) The expression levels of both genes were analyzed and compared using RT-qPCR.

To identify specific genes targeted by tangeretin in *F. verticillioides*, the differences of DEGs were compared and analyzed across various treatment groups. Moreover, the study incorporated resveratrol treatment as a comparative measure to delineate individual gene functional enrichments and to identify unique genes affected by tangeretin (Figure 6A). KEGG pathway enrichment analysis revealed that MAPK signaling pathway (fvr04011; Figure S4) was significantly enriched in the tangeretin treatment group compared to the stress-free group, while no obvious enrichment was observed in resveratrol treatment. This implies that tangeretin-induced stress, which correlates with a decrease in fumonisin production, may be linked to the MAPK signaling pathway. Furthermore, through a comprehensive analysis of the functions of the DEGs, two key genes, *FVEG_03055* and *FVEG_04238*, were ultimately identified as playing key roles in the antifungal and anti-mycotoxigenic activity of tangeretin (Figure 6B). These genes encode an NmrA- and HSCARG-like protein (designated as Fv_Tan1) and a multidrug resistance transporter (designated as Fv_Tan2), respectively. The relative transcriptional levels of these genes were further validated using RT-qPCR (Figure 6C).

Phylogenetic analysis showed that the Fv_Tan1 protein and its homologs in other *Fusarium* species closely relate to proteins found in *A. niger* and *A. flavus* but not those in *Penicillium expansum* (Figure S5). In contrast, Fv_Tan2 displayed high similarity with PEX2_100930 from *P. expansum* MD-8. Fv_Tan1 was predicted with other potential interaction proteins, whose expression showed no significant variation in *F. verticillioides* under tangeretin stress (Figure S6A; Table S3). Despite Fv_Tan1’s conservation within the NmrA-like regulator superfamily, it does not appear to be significantly involved with nitrogen metabolism as implied by protein–protein interaction analyses involving the nitrogen metabolism regulator AreA (FVEG_02033). Accordingly, AreA is more likely to interact with another NmrA-like regulator FVEG_07953 from the analysis of AreA interaction proteins (Figure S6B). Nevertheless, the expression of AreA and FVEG_07953 exhibited steadily in *F. verticillioides* with or without tangeretin treatment (Table S2). Furthermore, the presence of a conserved HSCARG domain in Fv_Tan1 suggests a role as an NADPH sensor, indicating a distinct mechanism of action from typical NmrA-like regulators with AreA. Moreover, the transporter Fv_Tan2 was considered to have 12 transmembrane helices potentially exerting xenobiotic resistance (Figure S7).

**Figure 7:**
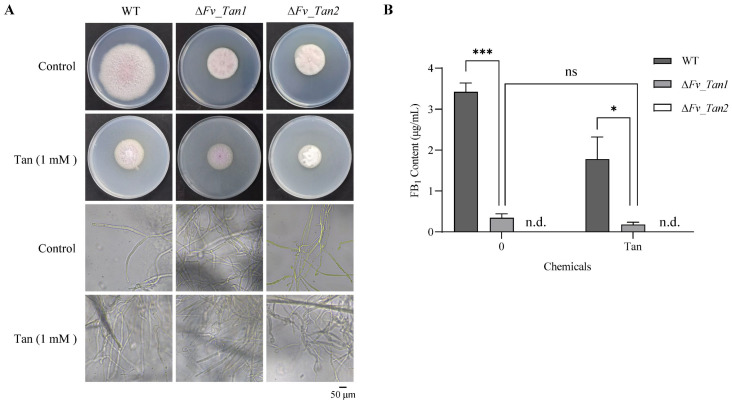
Effect of gene deletion on fungal growth and mycotoxin production. (**A**) Hyphal extension and microscopic observations in mutants ∆*Fv_Tan1* and ∆*Fv_Tan2*, showing notably slower growth, particularly in ∆*Fv_Tan1*. (**B**) Two mutant strains demonstrated a decrease in FB_1_ production when cultured in PDB medium, regardless of the presence of tangeretin stress. However, the FB_1_ production of the strain ∆*Fv_Tan1* in response to tangerentin did not exhibit a significant change compared to the control. Statistical significance of differences in FB_1_ levels between groups was denoted as * *p* < 0.05 and *** *p* < 0.001. The designation ‘ns’ denotes no significant difference between groups. Absence of detectable fumonisin production in the ∆*Fv_Tan2* mutant was labeled as ‘n.d.’ (not detected).

To confirm their roles in fungal growth and fumonisin biosynthesis in *F. verticillioides*, both genes were individually disrupted via homologous recombination (Figure S8). The hyphal growth of the gene-disrupted mutants became significantly slower than the wild-type strain of *F. verticillioides* (Figure 7A). Correspondingly, microscopic analysis revealed that *Fv_Tan1* could be responsible for the maintenance of mycelial integrity, as disruptions led to irregular growth and exacerbated structural anomalies under tangeretin stress condition. Conversely, deletion of *Fv_Tan2* caused no obvious influence of mycelial structure, suggesting a distinct role from *Fv_Tan1*. Overall, this analysis confirms the involvement of both genes in fungal growth, with *Fv_Tan1* specifically important for maintaining normal hyphal structure, highlighting the nuanced genetic impact of tangeretin on *F. verticillioides*.

Regarding mycotoxin production, the individual deletion profoundly affected the marked reduction of FB_1_ production compared to the wild-type strain (Figure 7B). Notably, the mutant deletion of *Fv_Tan2* halted fumonisin production entirely. In addition, the mutant without *Fv_Tan1* changed no obvious fluctuation in FB_1_ production when subjected to tangeretin stress. This possibly suggests that loss of *Fv_Tan1* may lessen the impact of tangeretin on mycotoxin production.

## 4. Discussion

Fumonisins are sphingosine-like mycotoxins linked with a variety of toxicities, including neurotoxicity, immunotoxicity and even carcinogenicity, by acting on sphingolipid metabolism, oxidative stress and apoptosis pathways [6,42,43]. *F. verticillioides* is the predominant fumonisin producing phytopathogen in maize. Plant-derived compounds are being explored as sustainable alternatives to the use of fungicides for the mitigation of pathogenic disease and fumonisin contamination. In this study, tangeretin was identified and evaluated for its potential to inhibit fumonisin production by *F. verticillioides*. While previous research has primarily focused on tangeretin’s antimicrobial properties against pathogens, such as respiratory syncytial virus [44], *Phytophthora citrophthora* [45], and *Magnaporthe oryzae* [46], this study is the first to investigate its antifungal and antimycotoxigenic potential against *F. verticillioides*. Our findings confirm its efficacy in inhibiting fumonisin production, thereby enhancing food safety prospects. This research establishes a theoretical foundation and underscores the practical value of tangeretin in managing fumonisin contamination. Notably, considering the neurotoxicity associated with fumonisins and the neuroprotective properties of tangeretin, this compound might also have therapeutic potential in mitigating fumonisin-induced neurotoxicity. To effectively address fumonisin control, future research should focus on improving the bioavailability of tangeretin. Nanoencapsulation presents a promising approach for enhancing the deployment of tangeretin in food safety applications, potentially leveraging green nanotechnology for efficient and cost-effective food preservation [47].

Tangeretin has been previously revealed to mitigate rice blast and inhibit the pathogenicity of *M. oryzae* by influencing redox signaling and ferroptosis processes [46]. A comparable mechanistic study in *F. verticillioides* revealed significant alterations in antioxidant activity, as evidenced by shifts in antioxidant enzymatic activities and H_2_O_2_ content. GO enrichment analysis indicated a notable delay in the transcription of oxidative stress-related DEGs. Specifically, DEGs associated with peroxidase activity (GO:0004601) exhibited lower transcription levels, underscoring a substantial disruption in the redox balance, consistent with findings in *M. oryzae* [46]. In contrast, the transcription levels of two genes (*FVEG_00444* and *FVEG_11478*) encoding NADPH oxidase (Nox) did not show significant variation, differing from observations in *M. oryzae* where Nox-mediated lipid peroxidation was targeted by overexpressed *Nox* genes. This suggests pathogen-specific effects of tangeretin, which may interact differently with the molecular targets in *F. verticillioides*. In addition, peroxisomes are essential for the cellular oxidative homeostasis, infection, virulence as well as mycotoxin biosynthesis in phytopathogenic fungi [48,49]. PEX genes are involved in peroxisome biogenesis [50,51]. Components of the peroxisomal docking/translocation module (DTM) crucial for peroxisome biogenesis—such as FvPex5 (FVEG_01301) [15], FvPex8 (FVEG_05423), FvPex13 (FVEG_00529), FvPex14 (FVEG_03847), and FvPex33 (FVEG_11334) [16]—play roles in redox homeostasis and fumonisin production in *F. verticillioides*. Despite this, the transcription of these genes did not significantly change under tangeretin stress. Similarly, the Atf1 orthologous bZIP-type transcription factor FvAtfA (FVEG_02866) [14], which is involved in the modulation of oxidative balance, virulence, and mycotoxin biosynthesis, also showed no differential expression in response to tangeretin. This suggests that tangeretin may target other genes to influence redox homeostasis and fumonisin biosynthesis that warrants further investigation.

In contrast to resveratrol, tangeretin not only suppresses hyphal growth but also effectively inhibits fumonisin production. Comparative transcriptome analysis between tangeretin- and resveratrol-treated groups identified unique tangeretin-responsive DEGs implicated in mycotoxin regulation in *F. verticillioides*. The differences in enriched KEGG pathway demonstrated that tangeretin significantly interfered the MAPK signaling pathway, while resveratrol does not. The correlation between mycotoxin production and the MAPK pathway suggests that tangeretin may hinder mycotoxin accumulation through MAPK cascade. Furthermore, an NmrA- and HSCARG-like protein (Fv_Tan1) and an MDR transporter (Fv_Tan2) emerged as promising molecular targets responsive to tangeretin. The functions of these genes positively influence both fungal growth and fumonisin production. Notably, a mutant with *Fv_Tan1* deletion showed no significant effect in mycotoxin production in response to tangeretin. Bioinformatic analysis of Fv_Tan1 indicates that it does not directly interact with nitrogen metabolism regulator AreA, as previously speculated. Instead, AreA is believed to interact directly with nitrogen metabolism repressor FVEG_07953, due to its high similarity with NmrA (XP_041146541.1) from *A. flavus*, which plays crucial roles in radial growth, aflatoxin production, and stress responses [52]. Additionally, NmrA-like proteins have been identified to play a significant role in oxidative stress responses [53,54,55]. Further exploration of gene targets revealed that the conserved domain of Fv_Tan1 aligned with that of an HSCARG-like protein, which act as an NADPH sensor, suggesting a potential relationship with redox modulation. Protein interaction analysis indicates Fv_Tan1 likely interacts with many NADH-ubiquinone oxidoreductase involved in the mitochondrial respiratory chain. During the gradual reduction process of oxygen (O_2_) to water by this chain, electron leakage can lead to the formation of ROS [56]. This suggests that Fv_Tan1’s role in mycotoxin biosynthesis may be linked to its regulation on redox homeostasis. In conclusion, tangeretin inhibits fumonisin biosynthesis by disrupting normal MAPK signaling pathways and targeting the NmrA- and HSCARG-like protein Fv_Tan1. By elucidating the modes of action, this study enhances our understanding of how plant-derived compounds, like tangeretin, can inhibit mycotoxin production and suggests new avenues for developing effective mycotoxin management strategies.

## 5. Conclusions

Tangeretin, known for its antioxidant, anti-inflammatory and anti-tumor properties, has also demonstrated notable antifungal and anti-fumonisin effects. It significantly downregulates the expression of DEGs associated with hyphal growth, conidial development, and pathogenesis, thereby disrupting redox regulation. Additionally, tangeretin restricts substrate availability for fumonisin biosynthesis by interfering with alanine metabolism, pyruvate metabolism, fatty acid degradation and sphingolipid metabolism. Furthermore, it may directly act on the biosynthetic gene cluster responsible for fumonisin production. The compound also disrupts the MAPK signaling pathway and targets a downstream NmrA- and HSCARG-like protein (Fv_Tan1). Gene deletion of Fv_Tan1 causes a significant reduction in hyphal growth and fumonisin level. Notably, tangeretin exerts minimal influence on mycotoxin production in the mutant lacking this gene, underscoring the specificity of its target interaction. This study provides a theoretical foundation and emphasizes the potential application of tangeretin in controlling fumonisin contamination.

## Data Availability

The sequence data have been deposited in the Genome Sequence Archive [26] in the National Genomics Data Center [27], China National Center for Bioinformation/Beijing Institute of Genomics, and the Chinese Academy of Sciences (GSA: CRA017074) that are publicly accessible at https://ngdc.cncb.ac.cn/gsa accessed on 17 June 2024.

## Supplementary Materials

The following supporting information can be downloaded at: https://www.mdpi.com/article/10.3390/jof11040313/s1, Figure S1: Suppressive effects of various plant-derived compounds on fungal growth; Figure S2: DEG count comparison from groups treated with tangeretin, resveratrol, or control; Figure S3: GO enrichment analysis from *F. verticillioides* transcriptomes under tangeretin stress; Figure S4: Comparison of KEGG pathway enrichment; Figure S5: Phylogenetic analysis of FVEG_03055 (Fv_Tan1; A) and FVEG_04238 (Fv_Tan2; B) using MEGA 11; Figure S6: Potential interaction proteins with FVEG_03055 (Fv_Tan1; A) and nitrogen metabolism regulator AreA (FVEG_02033; B); Figure S7: Protein transmembrane structure prediction of FVEG_04238 (Fv_Tan2); Figure S8: Construction of gene disruption mutants; Table S1: The list of primer pairs in this work; Table S2: Gene differential expression of Fusarium verticillioides treated with tangeretin (Tan), resveratrol (Res) or control (C); Table S3: Potential interaction proteins of FVEG_03055 (Fv_Tan1) by STRING Database.
