# Supplementary material for: Tangeretin Suppresses Fumonisin Production by Modulating an NmrA- and HSCARG-like Protein in Fusarium verticillioides
Source: J Fungi (Basel). 2025 Apr 15;11(4):313. doi: 10.3390/jof11040313 (PMC12028961; doi:10.3390/jof11040313)
Supplement: Supplementary file 1 [file jof-11-00313-s001.zip › jof-3548562-supplementary-Supplementary material.pdf]

Journal of *Fungi*

MDPI

Supplementary Materials

Tangeretin Suppresses Fumonisin Production by Modulating an NmrA- and HSCARG-Like Protein in *Fusarium verticillioides*

Liuqing Wang [1], Wenlei Zhai [1], Dongmei Jiang [1], Nan Jiang [1], Jiaqi Yan [2], Haoyun Jiang [1] and Meng Wang [1,*]

1 Institute of Quality Standard and Testing Technology, Beijing Academy of Agriculture and Forestry Sciences, No. 9 Middle Road of Shuguanghuayuan, Haidian District, Beijing 100097, China; wangliuqing@baafs.net.cn (L.W.); zhaiwl@iqstt.cn (W.Z.); jiangdongmei@baafs.net.cn (D.J.); jiangnan@baafs.net.cn (N.J.); 13240034978@163.com (H.J.)
2 College of Horticulture, China Agricultural University, No. 2 Yuanmingyuan West Road, Haidian District, Beijing 100193, China; yanjiaqi@cau.edu.cn
* Correspondence: wangm@iqstt.cn

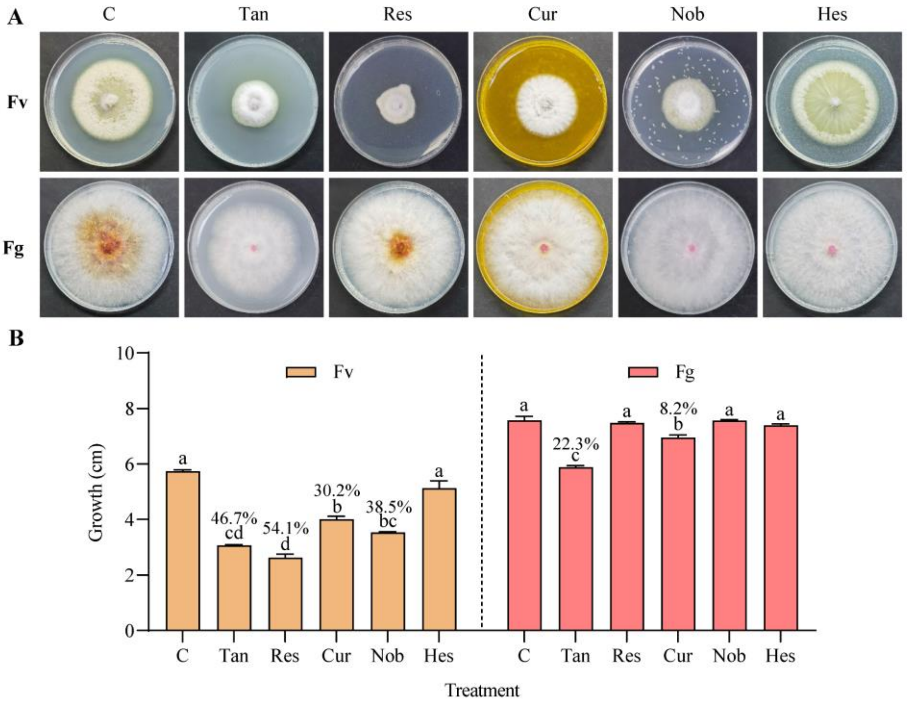

Figure S1. Suppressive effects of various plant-derived compounds on fungal growth. *Fusarium verticillioides* (Fv) and *F. graminearum* (Fg) were each treated with 1 mM of tangeretin (Tan), resveratrol (Res), curcumin (Cur), nobiletin (Nob), and hesperidin (Hes). Inhibitory impact was evaluated by hyphal growth extension.

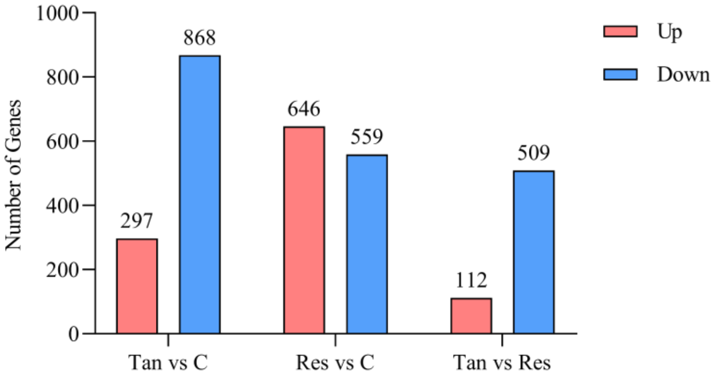

Figure S2. DEG count comparison from groups treated with tangeretin, resveratrol, or control.

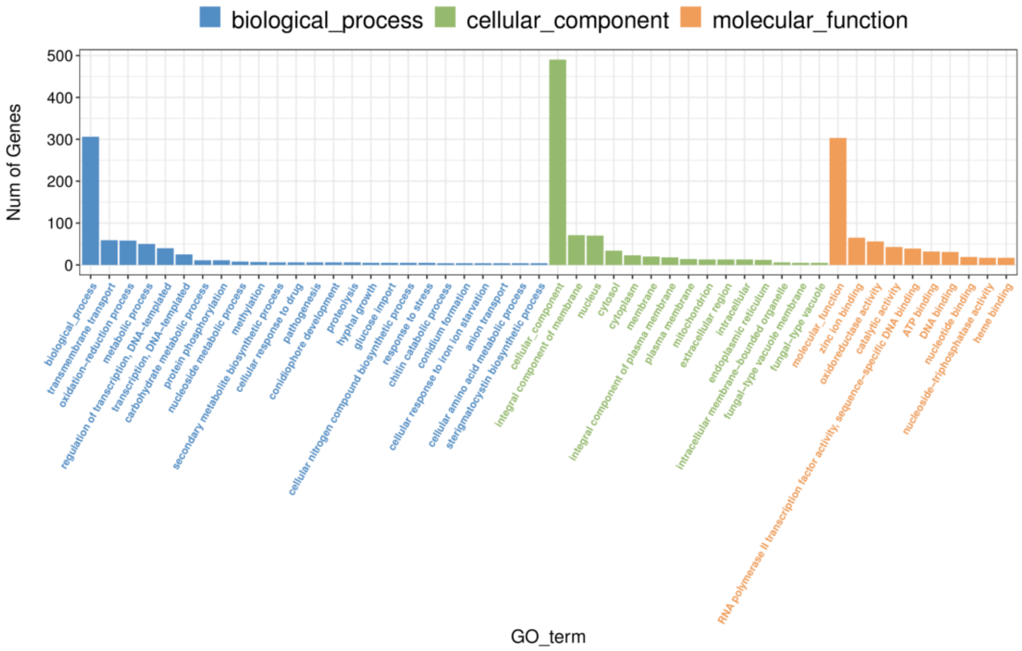

Figure S3. GO enrichment analysis from *F. verticillioides* transcriptomes under tangeretin stress. Analysis of biological processes, cellular components, and molecular functions based on DEG counts in each GO category.

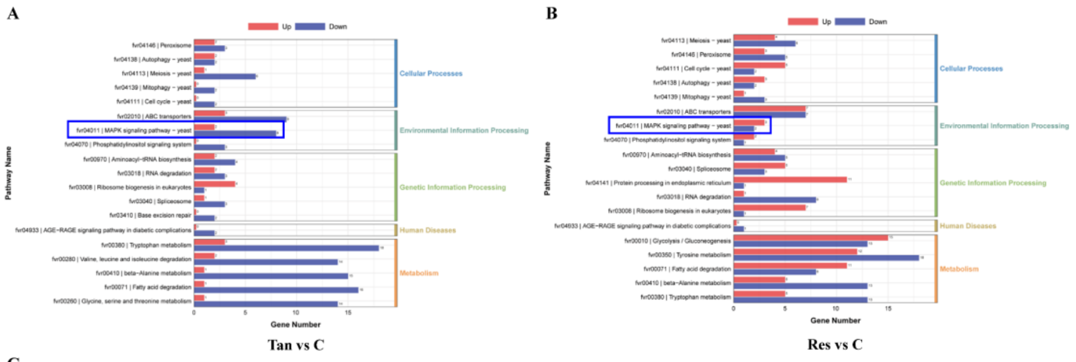

C

Tan_VS_C.KEGG_Enrichment											
Pathway_ID	Pathway_Name	S	TS	B	TB	Rich.Factor	P.value	Sig.Sign.P	Q.value	Sig.Sign.Q	ZScore
fvr04011	MAPK signaling pathway	10	315	120	14916	0.0833	0.000228344	***	0.00077474	***	-3.103761159
**Res_VS_C.KEGG_Enrichment**											
Pathway_ID	Pathway_Name	S	TS	B	TB	Rich.Factor	P.value	Sig.Sign.P	Q.value	Sig.Sign.Q	ZScore
fvr04011	MAPK signaling pathway	5	369	120	14916	0.0417	0.176609253	ns	0.237028208	ns	-6.207522318

Figure S4. Comparison of KEGG pathway enrichment. This result was evaluated from different treatments with tangeretin (Tan; A) and resveratrol (Res; B), compared to the control. The MAPK signaling pathway (fvr04011) demonstrated significant enrichment in the tangeretin treatment group (*** *P*<0.001); conversely, no obvious enrichment was detected in the resveratrol treatment group (C).

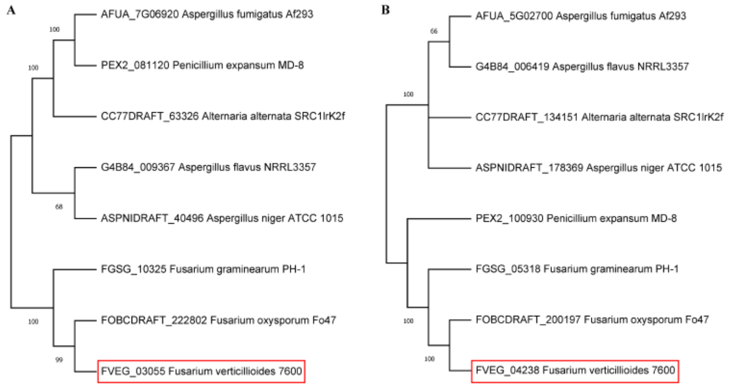

Figure S5. Phylogenetic analysis of FVEG_03055 (Fv_Tan1; A) and FVEG_04238 (Fv_Tan2; B) using MEGA 11. This analysis utilized amino acid sequences of homologous proteins from mycotoxin-producing fungi. The Neighbor-Joining tree was constructed employing the bootstrap method, with 500 replications based on the Poisson model.

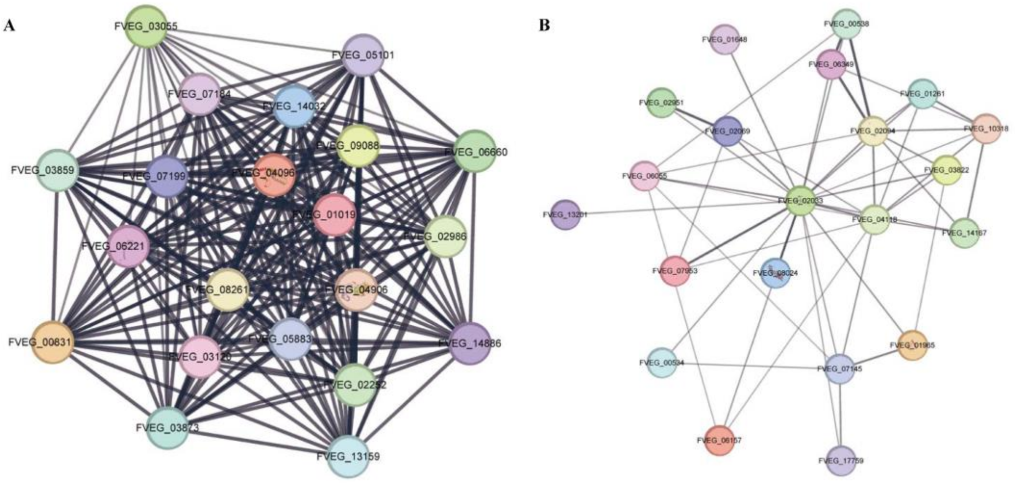

Figure S6. Potential interaction proteins with FVEG_03055 (Fv_Tan1; A) and nitrogen metabolism regulator AreA (FVEG_02033; B). Potential proteins were analyzed using the STRING database. A medium confidence interaction score of 0.400 was established as the minimum threshold, and a maximum of 20 interacting proteins were displayed based on their corresponding scoress.

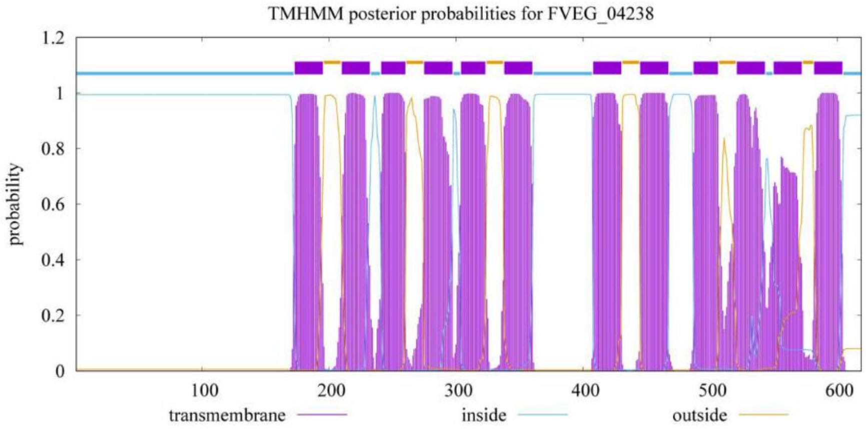

Figure S7. Protein transmembrane structure prediction of FVEG_04238 (Fv_Tan2). Transmembrane helices were pre-dicted based on amino acid sequence of Fv_Tan2 by TMHMM-2.

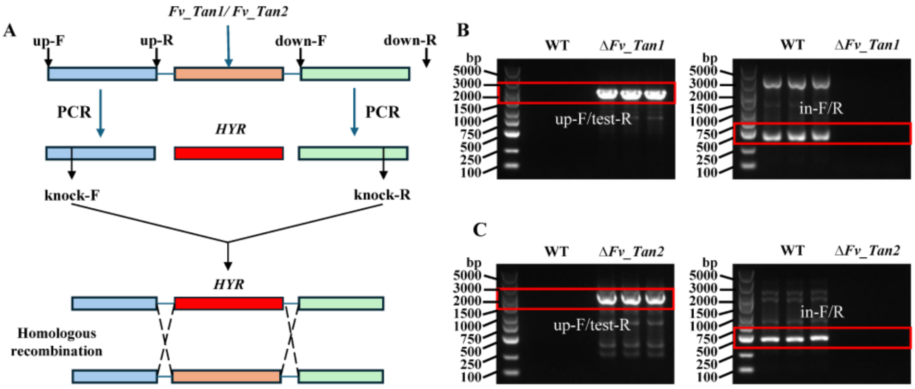

D

slope		inter		Ct hph		Ct β-tubulin		copy number	
hph	β-tubulin	hph	β-tubulin	WT	Δ*Fv_Tan1*	WT	Δ*Fv_Tan1*	WT	Δ*Fv_Tan1*
-1.917	-3.470	35.953	30.777	34.037	29.867	20.400	19.853	0.010	1.064

E

slope		inter		Ct hph		Ct β-tubulin		copy number	
hph	β-tubulin	hph	β-tubulin	WT	Δ*Fv_Tan2*	WT	Δ*Fv_Tan2*	WT	Δ*Fv_Tan2*
-2.257	-3.323	34.728	29.780	34.870	28.903	22.113	21.457	0.004	1.193

Figure S8. Construction of gene disruption mutants. The genes *FvEG_03055* (*Fv_Tan1*) and *FvEG_04238* (*Fv_Tan2*) were individually disrupted via homologous recombination (A). The resulting transformants were identified by PCR, gel electrophoresis and qPCR. Among them, the primer pairs (up-F/test-R) were designed to amplify the recombinant sequences of the mutant Δ*Fv_Tan1* (B) and Δ*Fv_Tan2* (C) containing the upstream of the corresponding gene and partial sequence of hygromycin resistance gene. Otherwise, the primer pair (in-F/R) was designed to amplify the original sequences of *Fv_Tan1* and *Fv_Tan2* that was deleted by homologous recombination. Furthermore, the copy number of the integrated sequences of Δ*Fv_Tan1* (D) and Δ*Fv_Tan2* (E) was determined by qPCR avoiding the sequences integrated elsewhere in the genome, as referenced in Zhang et al. [1].

Reference

1. Zhang, J.; Zhu, L.; Chen, H.; Li, M.; Zhu, X.; Gao, Q.; Wang, D.; Zhang, Y. A polyketide synthase encoded by the gene An15g07920 is involved in the biosynthesis of ochratoxin A in *Aspergillus niger*. *J. Agric. Food Chem.* **2016**, *64*, 9680–9688, doi:10.1021/acs.jafc.6b03907.
